# Intermittent administration of a fasting-mimicking diet intervenes in diabetes progression, restores β cells and reconstructs gut microbiota in mice

**DOI:** 10.1186/s12986-018-0318-3

**Published:** 2018-11-20

**Authors:** Siying Wei, Ruomei Han, Jingyu Zhao, Shuo Wang, Meiqin Huang, Yining Wang, Yan Chen

**Affiliations:** CAS Key Laboratory of Nutrition, Metabolism and Food Safety, Shanghai Institute of Nutrition and Health, Shanghai Institutes for Biological Sciences, University of Chinese Academy of Sciences, Chinese Academy of Sciences, 320 Yueyang Rd, Shanghai, 200031 China

**Keywords:** Diabetes, Intermittent fasting, Fasting-mimicking diet, Beta cells, Fatty liver, Gut microbiota

## Abstract

Fasting and especially intermittent fasting have been shown to be an effective intervention in many diseases, such as obesity and diabetes. The fasting-mimicking diet (FMD) has recently been found to ameliorate metabolic disorders. To investigate the effect of a new type of low-protein low-carbohydrate FMD on diabetes, we tested an FMD in *db/db* mice, a genetic model of type 2 diabetes. The diet was administered every other week for a total of 8 weeks. The intermittent FMD normalized blood glucose levels in *db/db* mice, with significant improvements in insulin sensitivity and β cell function. The FMD also reduced hepatic steatosis in the mice. Deterioration of pancreatic islets and the loss of β cells in the diabetic mice were prevented by the FMD. The expression of β cell progenitor marker Ngn3 was increased by the FMD. In addition, the FMD led to the reconstruction of gut microbiota. Intermittent application of the FMD increased the genera of *Parabacteroides* and *Blautia* while reducing *Prevotellaceae, Alistipes* and *Ruminococcaceae.* The changes in these bacteria were also correlated with the fasting blood glucose levels of the mice. Furthermore, intermittent FMD was able to reduce fasting blood glucose level and increase β cells in STZ-induced type 1 diabetic mouse model. In conclusion, our study provides evidence that the intermittent application of an FMD is able to effectively intervene in the progression of diabetes in mice.

**Electronic supplementary material:**

The online version of this article (10.1186/s12986-018-0318-3) contains supplementary material, which is available to authorized users.

## Introduction

Animals, including humans, have evolved physical adaptations to periodic long-term fasting and short-term feasting due to the scarcity of available food in the environment. Some animals become dormant during extended periods of food deprivation. While some animals hibernate during extended periods of food deprivation, a principle adaptation to fasting observed in humans is the development of energy depot organs, such as fat and the liver, to efficiently preserve energy acquired during feeding for survival. Not surprisingly, fasting has long been considered and proven to be the best intervention in metabolic disorders associated with obesity and can extend the lifespan of various organisms, from worms to mammals [1]. However, considering the periodic nature of the fasting-feeding cycle to which animals have adapted during evolution, periodic fasting (PF) or intermittent fasting (IF) have been proposed to improve metabolic health and aging [2–4]. In 1946, Carlson and Hoelzel first reported that IF was able to prolong the lifespan of rats [5]. Also, in 1962, Duncon et al found that IF was able to control obesity in humans [6]. An early study also indicated the beneficial effects of IF on blood glucose levels, insulin resistance, cardiovascular function, and brain function [7]. IF with complete fasting every other day extended the lifespan and increased the resistance of the brain to metabolic and excitotoxic insults [8]. Similarly, alternate-day fasting in mice led to a reduction in blood glucose levels and an increase in the resistance of neurons in the brain to excitotoxic stress, independent of total caloric intake [9]. In addition, alternate-day fasting in obese women significantly reduced body weight, fat mass, blood cholesterol concentration, and blood triglyceride concentration [10]. Time-restricted feeding (TRF) had a protective effect on postmenopausal obesity in mice [11]. TRF was also able to attenuate metabolic disorders in mice, and most importantly, the protective effects were maintained even when TRF was temporarily interrupted by ad libitum access to food during weekends [12], indicating a potentially promising regimen for application in to human lifestyles. The mechanisms underpinning the apparent beneficial effects of IF were recently shown to be associated with its ability to promote browning of white adipose tissues via alterations of gut microbiota [13]. However, a recent clinical trial indicated that although alternate-day fasting reduced body weight, it did not produce superior adherence, weight loss, weight maintenance, or cardioprotection when compared to continuous daily calorie restriction in obese adults [14], indicating that future studies are still needed to provide optimal IF regimens for intervention in obesity and other related diseases.

To promote compliance with IF in humans, Dr. Longo’s laboratory has introduced the concept of a fasting-mimicking diet (FMD) to replace simple fasting [15]. FMD administration for 4 days bimonthly in middle-aged mice extended longevity, lowered visceral fat and reduced cancer incidence [15]. Periodic 3-day cycles of FMD were able to ameliorate demyelination and symptoms in a murine experimental autoimmune encephalomyelitis model [16]. A pilot study using an FMD was potentially effective in the treatment of patients with relapsing-remitting multiple sclerosis patients [16]. A combination of chemotherapy and an FMD was also found to reduce the progression of breast cancer and melanoma by promoting T cell-mediated cytotoxicity [17]. Recently, a human study with 100 healthy subjects demonstrated that an FMD low in calories, sugar, and protein but high in unsaturated fat had beneficial effects on body mass index, blood pressure, fasting glucose, IGF-1, triglycerides, total and low-density lipoprotein cholesterol, and C-reactive protein in subjects at risk for disease compared to those in subjects who were not at risk [18]. However, one of the most remarkable studies was that of Cheng et al., who discovered that an FMD was able to promote β cell generation, restore insulin secretion and recover glucose homeostasis in mouse models of type 1 and type 2 diabetes [19], thus heralding the potential of the FMD to reverse the pathogenesis of diabetes.

In this study, we aimed to investigate whether a new type of low-protein low-carbohydrate FMD was able to intervene in type 2 diabetes in mice. In particular, we aimed to investigate whether intermittent administration of this FMD could restore the function of β cells that had been lost in *db/db* mice; and whether gut microbiota could contribute to the interventional effect of the FMD.

## Methods and materials

### Mouse model

Six-week-old male C57BL/ksJ-db (*db/db*) mice purchased from SLAC (Shanghai, China) were maintained in pathogen-free conditions and kept on a 12 h light/dark cycle at the Institute for Nutritional Sciences. All mice were weighed at the beginning of the study and randomly allocated to two groups: standard chow with free access to food and water (CTRL) and intermittent fasting with the FMD (~ 30% of the daily calorie intake of CTRL group) for 1 week, followed by ad libitum feeding for another week (FMD). For type 1 diabetes mouse model, low-dose streptozotocin (STZ) (40 mg/kg) was injected intraperitoneally for five consecutive days. The mice were weighed and fasted 8 h prior to STZ injection. STZ was dissolved in sodium citrate buffer (pH 4.5) and an equal volume of citrate buffer was injected into control mice. These experiments were conducted in accordance with the guidelines of the Institutional Animal Care and Use Committee of the Institute for Nutritional Sciences, Shanghai Institutes for Biological Sciences (SIBS), Chinese Academy of Sciences (CAS) with an approval number 2010-AN-8.

### Mouse fasting mimicking diet

The FMD (called Gembynear Nutrition Bar or Zhenbainian in Chinese) used in this study was kindly provided by the Beijing Winlife Research Institute of Nutrition, Health, Food Science, and Technology (Beijing, China). The composition and nutritional data of the FMD are given in Additional file 1: Tables S1 and S2. All mice were supplied with fresh food in the morning (9:00 a.m-10:00 a.m). The FMD mice generally consumed the supplied food within the first few hours.

### Mice fecal sample collection

All mice were caged individually. Fresh fecal samples of all mice were collected at 14:00 p.m.~ 15:00 p.m. to minimize possible circadian effects. Samples were collected into empty microtubes on ice and immediately stored at − 80 °C for future use.

### Blood glucose and insulin measurement

Mice were fasted for 6 h (9:00 a.m. ~ 15:00 p.m.) before blood glucose measurements. Blood glucose was measured through the tail vein using the OneTouch UltraEasy Blood Glucose Monitoring System (Lifescan, Milpitas, CA, USA). Serum insulin levels were determined by a murine enzyme-linked immunosorbent assay (Shanghai Enzyme-linked Biotechnology Co., Shanghai, China). Whole blood was withdrawn from the eyeball, and plasma was separated by centrifugation at 3,000 rpm for 15 min in EDTA-K2-treated microtubes (Kangjian Medical, China). The homeostatic model assessment (HOMA) was used to quantify insulin resistance (HOMA-IR) and β cell function (%B). HOMA-IR was calculated using the following formula: HOMA-IR = (fasting glucose × fasting insulin)/22.5. HOMA %B was calculated using the following formula: HOMA-%B = (20 × fasting insulin)/(fasting glucose - 3.5) %.

### Glucose tolerance testing (GTT) and insulin tolerance testing (ITT)

Mice were caged individually and fasted for 4 h for ITT (morning fasting) and fasted overnight for GTT. Glucose (2 g/kg) or insulin (2 unit/kg) was injected intraperitoneally. Blood glucose levels were measured at 0, 15, 30, 60, and 90 min after each injection.

### Measurement of serum and hepatic parameters

Mice were euthanized, and blood was immediately collected from the orbital sinus into EDTA-K2-treated microtubes (Kangjian Medical, China). Then, the microtubes were centrifuged at 3,000 rpm for 15 min, and the plasma supernatant was divided into 3 portions for different uses. All plasma samples, excluding those for immediate use, were stored at − 80 °C. Hepatic lipids were extracted with chloroform/methanol (2:1). Plasma levels of aspartate transaminase (AST) and alanine transaminase (ALT) were determined by an AST/ALT determination kit (ShenSuo UNF, China). Plasma and hepatic levels of triglycerides (TG) and total cholesterol (TC) were determined by colorimetric methods with the corresponding kits (ShenSuo UNF, China). All of these assays were performed according to the manufacturer’s instructions.

### Immunofluorescence analysis

Mouse tissues were fixed in 4% paraformaldehyde, dehydrated and embedded into paraffin. The tissues were sectioned into thick slices (4 μm), deparaffinized in xylene and rehydrated via a graded ethanol series (100%, 90%, 70%, 50%, and 30%) and water. The antigen was retrieved by heat treatment with 0.1 M citrate buffer (pH = 6.0), and the sections were blocked with blocking buffer (PBS + 1% normal goat serum+ 0.1% trixton-100). The following primary antibodies were used: anti-insulin (C27C9 from Cell Signaling Technology, Boston, MA, USA), anti-glucagon (ab10988 from Abcam, MA, USA), anti-Ngn3 (sc-374442 from Santa Cruz Biotechnology, Dallas, Texas, USA), and anti-Ki67 (550609 from BD Biosciences, New Jersey USA). Sections were incubated with primary antibodies in a humidified chamber overnight. After washing with PBS, the sections were incubated for 1 h at RT with secondary antibodies (Alexa Fluor 488 donkey anti-rabbit IgG, Alexa Fluor 546 donkey anti-mouse, dilution 1/500). All secondary fluorochrome-conjugated antibodies were purchased from Life Technologies (Eugene, OR, USA). The images of the stained sections were captured using a 40x objective with an LSM 510 confocal microscope (Zeiss, Jena, Germany).

### H&E staining of liver samples

Mouse livers were dissected and washed in PBS. All tissues were fixed in 4% paraformaldehyde for 48 h at room temperature, dehydrated and embedded into paraffin. Then, the tissues were sectioned into thick slices (4 μm) and stained with hematoxylin and eosin (H&E).

### RNA isolation, RT-PCR and real-time PCR

Mouse liver tissues were lysed in TRIzol reagent (Invitrogen, CA, USA). Total RNA was purified according to the manufacturer’s instructions, reverse transcribed, and synthesized to complementary DNA using a FastQuant RT Kit (with gDNase) (Tiangen Biotech Co., LTD, Beijing, China). Real-time PCR was conducted with the ABI Prism 7900 sequence detection system following the manufacturer’s recommendations (Applied Biosystems, CA, USA). Relative mRNA levels were quantified using the comparative ΔCT method and normalized to actin with the sequences of primers written in Additional file 1: Table S4.

### Analysis of gut microbiota

Fecal DNA extraction, PCR amplification, and 16 s rRNA sequencing were performed by Majorbio (Shanghai, China) and the data were analyzed as previously reported [20]. In brief, fecal DNA was extracted using the E.Z.N.A.® soil DNA Kit (Omega Biotek, Norcross, GA, U.S.) according to manufacturer’s instructions. The DNA concentration and purity was determined by a NanoDrop 2000 UV-vis spectrophotometer (Thermo Scientific, Wilmington, USA), and the quality was verified using 1% agarose gel electrophoresis. The V3-V4 regions were then amplified, and the PCR products generated were extracted from a 2% agarose gel and purified using the AxyPrep DNA Gel Extraction Kit (Axygen Biosciences, Union City, CA, USA). Purified products were pooled in equimolar quantities and paired-end sequenced (2 × 300) on an Illumina MiSeq platform (Illumina, San Diego, USA) according to the manufacturer’s protocols (Majorbio Bio-Pharm Technology Co. Ltd., Shanghai, China). Raw fastq files were demultiplexed, quality-filtered by Trimmomatic and merged by FLASH. Operational taxonomic units (OTUs) were clustered with a 97% similarity cutoff by using UPARSE (version 7.1 http://drive5.com/uparse/) and chimeric sequences were noted and deleted using UCHIME. The taxonomy of each 16S rRNA gene sequence was studied by the RDP Classifier algorithm (http://rdp.cme.msu.edu/) against the Silva (SSU123) 16S rRNA database using confidence threshold of 70%.

### Statistical analysis

All data were expressed as the mean ± SEM. Significant differences were assessed either by two-tailed Student’s *t*-tests or by one-way ANOVA followed by the Student-Newman-Keuls test where appropriate.

## Results

### Intermittent administration of FMD intervenes in the pathology of type 2 diabetes in *db/db* mice without significant changes in body weight

**Fig. 1:**
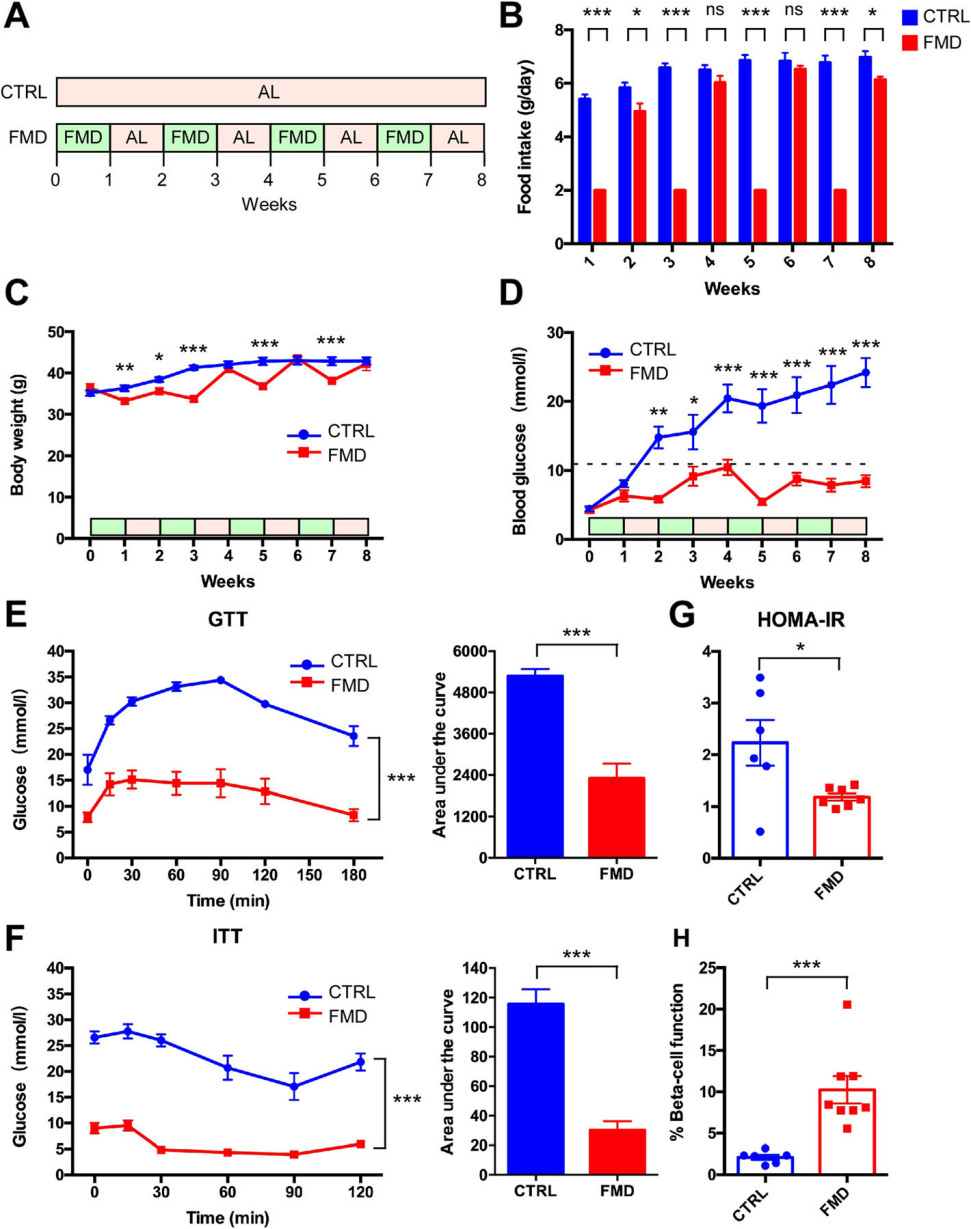
Intermittent FMD intervenes in type 2 diabetes in *db/db* mice. **a** Experimental scheme to determine effects of the FMD in T2D in leptin-receptor-deficient *db/db* mice. Six-week-old male *db/db* mice were assigned to two groups: control and FMD (*n* = 8 for each group). Each FMD cycle entails 7 days of FMD and 7 days of ad libitum feeding (AL). During free feeding, mice received a regular chow identical to that given prior to the FMD and that given to the ad libitum (AL) controls. **b** Food intake. **c** Body weight. **d** Blood glucose levels. Blood samples were collected on the last day of each cycle. Mice were fasted for 6 h (morning fasting) for blood glucose measurements (n = 8 for each group). A fasting blood glucose level of > 11.1 mmol/L was defined as diabetic (dotted line). **e**, **f** Glucose tolerance test and insulin tolerance test at week 9. The area under the curve is shown on the right. **g**, **h** Homeostatic model assessment (HOMA) of insulin resistance (IR) and steady-state β cell function (%B) at week 9. Data are expressed as the mean ± SEM, n = 8 per group. * *p* < 0.05, ** *p* < 0.01, *** *p* < 0.001, ns for nonsignificant

We first investigated whether the FMD was able to intervene in the progression of type 2 diabetes in *db/db* mice that are susceptible to the development of severe diabetes due to morbid obesity caused by a lack of leptin receptors. The FMD used in this study was vegetable-based, low in protein/carbohydrates and high in fat as compared to standard chow; and contained multiple vegetable ingredients without preservatives (Additional file 1: Tables S1-S3). The *db/db* mice were divided into two groups (*n* = 8 for each group) (Fig. 1a). The control group had ad libitum (AL) access to standard food. The FMD group was exposed to alternating FMD and ad libitum diet every other week. The FMD group had free access to standard food during the AL week. On average, food intake was 6.47 g/day in the control group. For the FMD, food intake was 2 g/day during the FMD week and 5.91 g/day during the AL week (Fig. 1b). The average energy intake was 22.13 kcal/day in the control group, 8.12 kcal/day during the FMD week and 17.75 kcal/day during the AL week in the FMD group. During the FMD weeks, except for the first week, the body weight of the FMD group was significantly lower than that of the control group (Fig. 1c). Notably, at the end the 8-week period, the body weights of the two groups were not significantly different from each other (Fig. 1c).

The elevated fasting blood glucose level in *db/db* mice was significantly reduced by the FMD. Starting from the beginning of the second week, the fasting blood glucose level was significantly lower in the FMD group than in the control group (Fig. 1d). A fasting blood glucose level of > 11.1 mmol/L was defined as diabetic. With this cut off criterion, the FMD completely intervenes in the pathology of type 2 diabetes in the mice. Both GTT and ITT tests performed at the end of the experiment indicated consistent significant improvements in glucose tolerance and insulin sensitivity after the FMD (Fig. 1e and f). Likewise, HOMA-IR was significantly reduced by the FMD in these mice (Fig. 1g). Meanwhile, the calculated β cell function was significantly elevated by the FMD (Fig. 1h). Collectively, these data indicate that intermittent administration of an FMD is able to drastically intervene in type 2 diabetes progression in the mice.

### Hepatic steatosis is improved by intermittent administration of an FMD

**Fig. 2:**
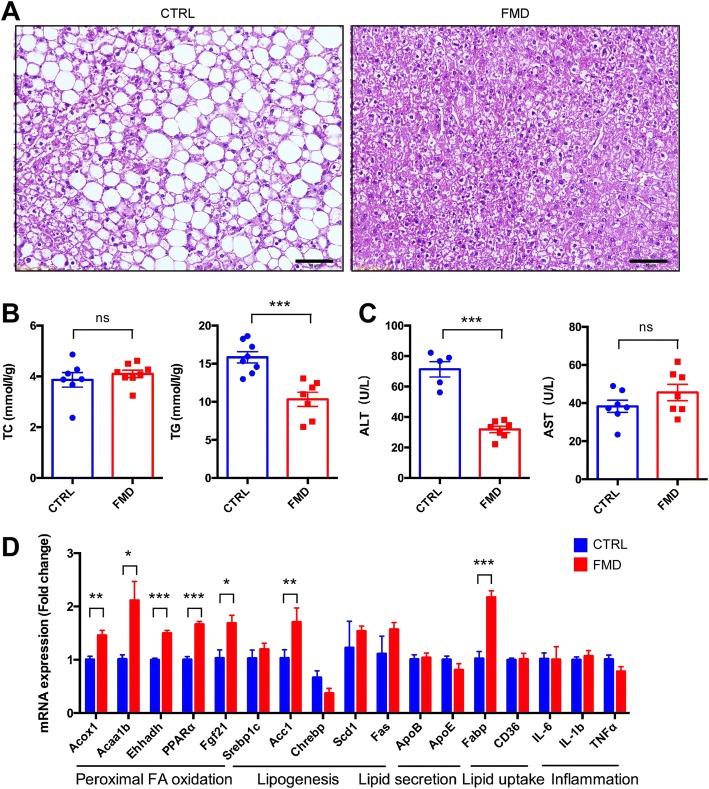
Intermittent FMD reduced hepatic steatosis in *db/db* mice. **a** Representative H&E staining of liver sections. Scale bar: 50 μm. **b** Levels of total triglycerides and total cholesterol in the liver. **c** Blood ALT and AST levels of the mice. **d** Quantitative RT-PCR results to detect expression of genes involved in peroxisomal fatty acid oxidation, lipogenesis, lipid secretion, lipid uptake, and inflammation. Mouse livers were used to isolate total RNA, followed by reverse transcription and real-time PCR. Data are expressed as the mean ± SEM, n = 8 per group. * *p* < 0.05, *** *p* < 0.001, ns for nonsignificant

Overt obesity developed in the *db/db* mice fed the control diet, which was accompanied by severe fatty liver, shown as the accumulation of lipid droplets in hepatocytes (Fig. 2a). However, the FMD markedly reduced the formation of fatty liver, as examined by histology (Fig. 2a). Consistently, the total glyceride (TG) level in the liver was significantly reduced by the FMD (Fig. 2b) without concomitant changes in the total cholesterol level (Fig. 2b). On the other hand, the elevation in the level of ALT, an indicator of liver damage, was significantly lowered by the FMD, although the AST level was not altered (Fig. 2c). These data therefore indicate that hepatic steatosis in *db/db* mice was reduced by the FMD. We also analyzed whether the FMD improved lipid metabolism in the liver. Quantitative RT-PCR of liver tissues revealed that the expression of some peroxisomal fatty acid oxidation-related genes, including peroxisomal acyl-coenzyme A oxidase 1 (Acox1), acetyl-coenzyme A acyltransferase 1 (Acaa1), enoyl-CoA hydratase and 3-hydroxyacyl CoA dehydrogenase (Ehhadh), and peroxisome proliferator-activated receptor α (PPARα), was upregulated by the FMD; while most other genes involved in lipogenesis, lipid secretion, lipid uptake, and inflammation were not significantly changed (Fig. 2d); indicating that the FMD improved hepatic steatosis mainly through promoting fatty acid oxidation.

### Loss of pancreatic islets and β cells in *db/db* mice was prevented by the FMD

**Fig. 3:**
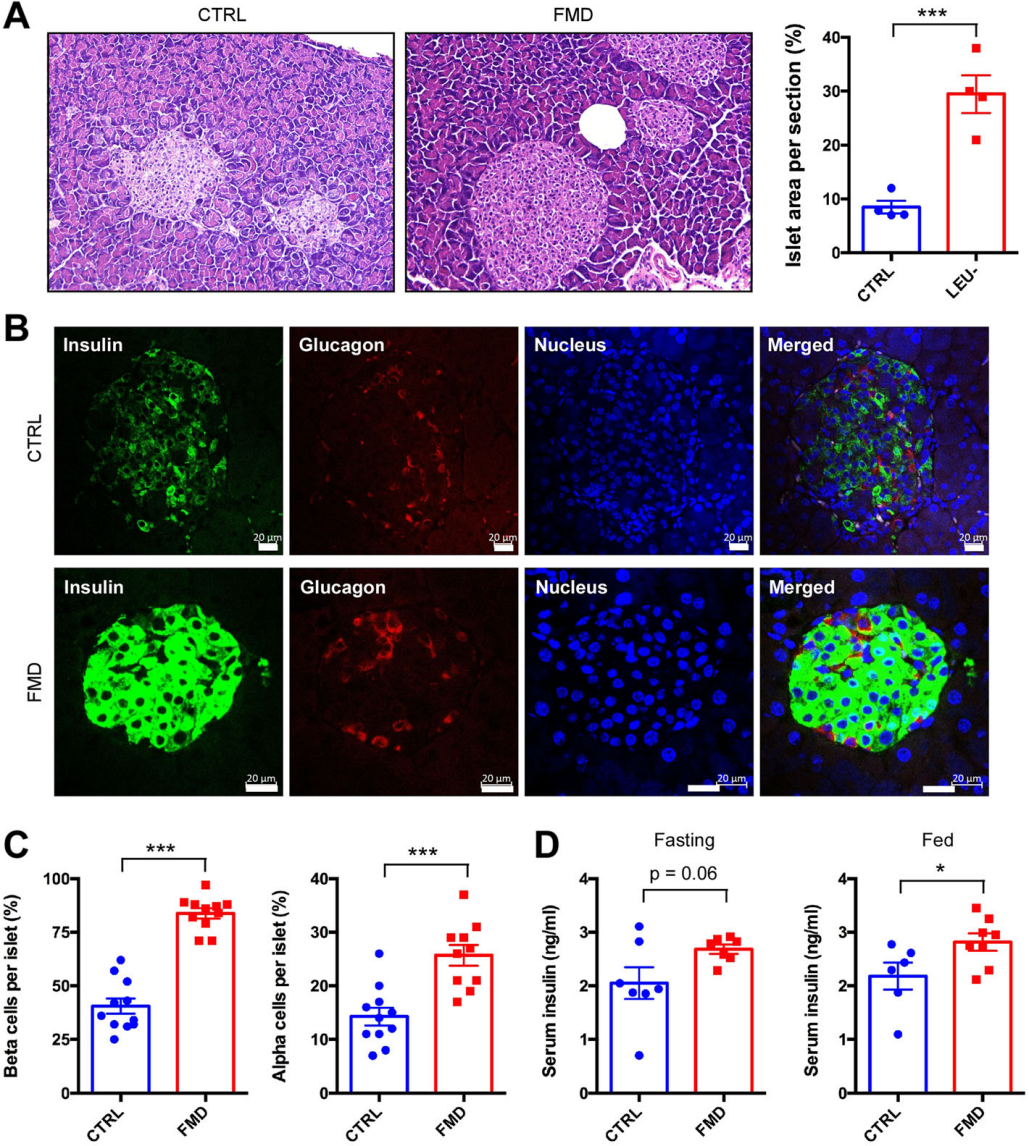
Intermittent FMD restores the cells of pancreatic islets in *db/db* mice. **a** Representative H&E staining of islets in pancreatic sections. Scale bar: 50 μm. The ratio of islet area in the pancreas sections is shown in the right panel. **b** Immunostaining of pancreatic sections to detect expression of insulin and glucagon. Scale bar: 20 μm. **c** Percentage of β cells and α cells per islet. **d** Serum insulin levels under fasting and fed state. Blood samples were collected at week 9. Mice were fasted for 6 h for blood glucose measurements or without fasting. Data are expressed as the mean ± SEM, n = 8 per group. *p < 0.05, *** < 0.001

We also investigated the potential effects of the FMD on β cells in the islets. As expected, at the end of the experiment the β cells in the *db/db* mice of the control group suffered from severe deterioration shown by deformed and irregular islets with few scattered β cells (Fig. 3a). However, the FMD completely restored the islet to a normal shape and density of β cells (Fig. 3a). Quantitation of the islets revealed that the numbers of both β cells and α cells were significantly elevated by the FMD (Fig. 3b). The recovery of islet function by the FMD was also reflected by an increase in blood insulin levels during fed conditions (Fig. 3c).

**Fig. 4:**
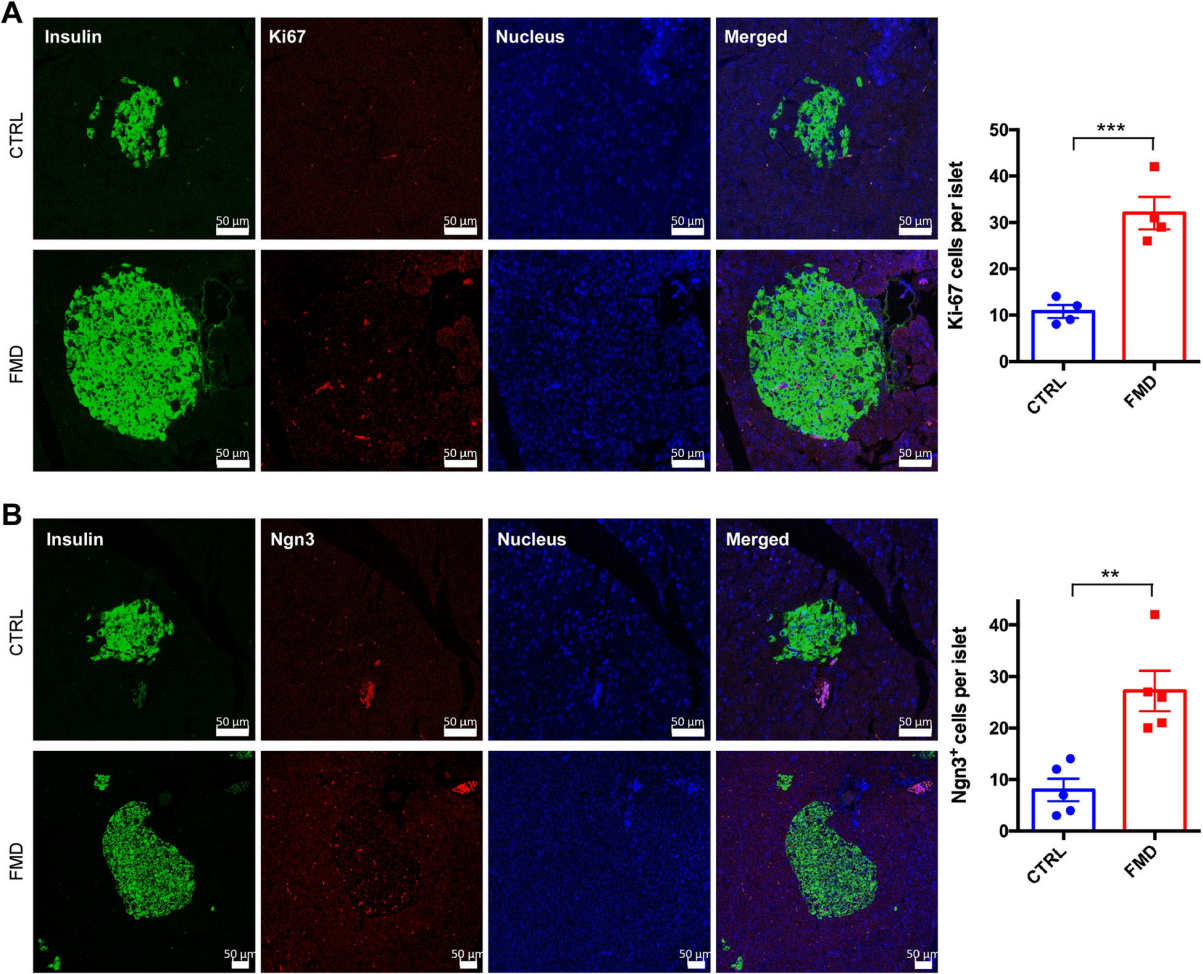
Intermittent administration of FMD increases β cell proliferation and Ngn3 expression in pancreatic islets. Representative immunofluorescence staining of pancreatic sections to detect Ki67 (**a**) and Ngn3 (**b**). Scale bar: 50 μm. The nucleus was stained with Hoechst 33342. Quantification of the images is shown in the right panel. Data are expressed as the means ± SEM. *** *p* < 0.001

We next investigated whether the increase in β cells in the mice was associated with changes in cell proliferation and expression of Ngn3, a marker for progenitors giving rise to β cells [19]. Immunofluorescent staining with the cell proliferation marker Ki67 showed that the rate of cell proliferation of β cells was significantly increased by the FMD (Fig. 4a). Furthermore, staining with Ngn3 in the islets was also significantly increased by the FMD (Fig. 4b). These data, therefore, indicate that the FMD can increase β cell proliferation and the number of β cell progenitors.

### Intermittent FMD alters the composition of the gut microbiota

**Fig. 5:**
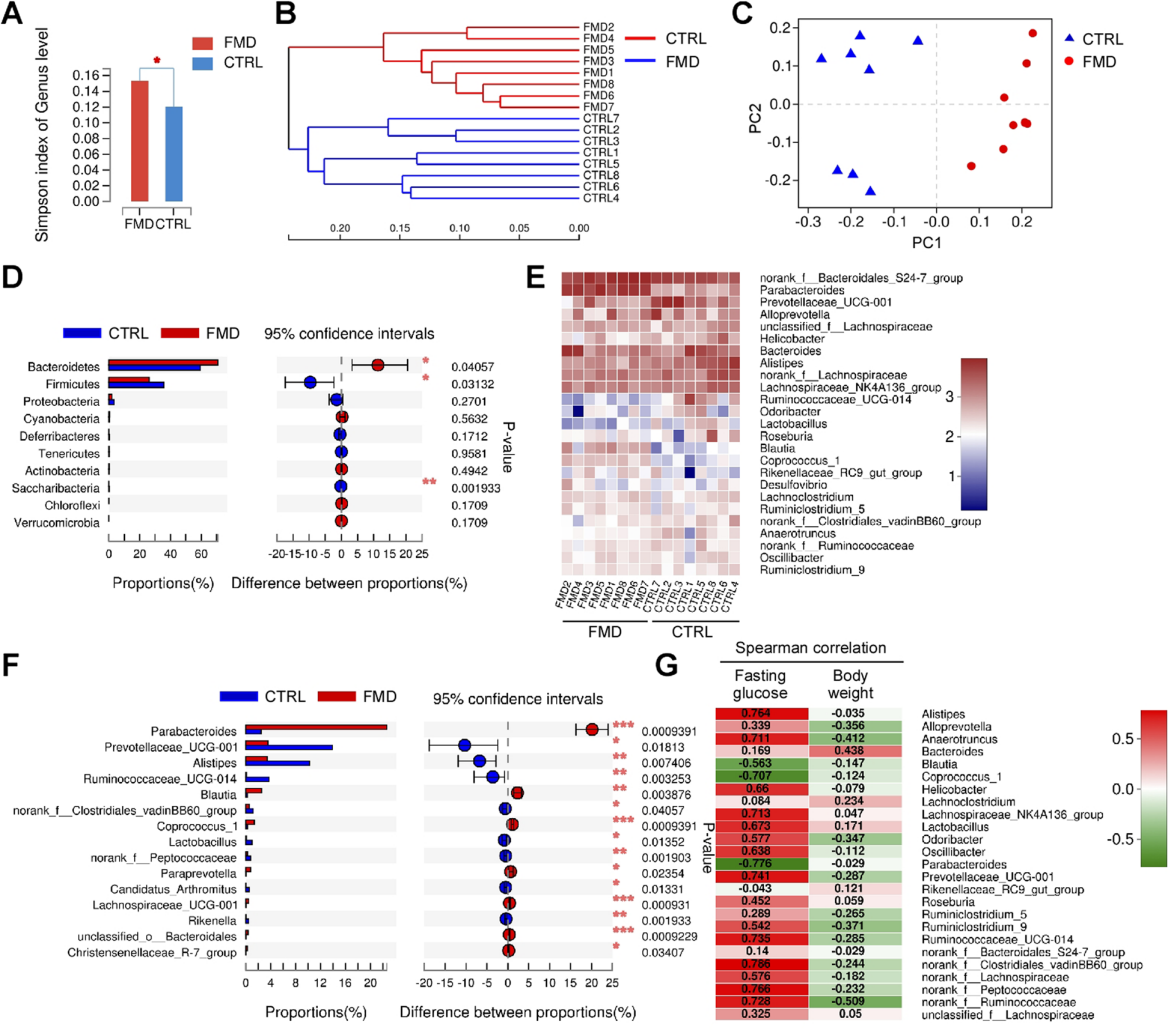
Intermittent FMD alters gut microbiota in *db/db* mice. **a** Simpson index of gut microbes at genus level. Data are expressed as the mean ± SEM, *n* = 8 per group. * *p* < 0.05. **b** Hierarchical clustering of gut microbiota at the genus level (*n* = 7 mice/group). **c** PCoA plot based on the relative abundance of microbiome at the genus level. **d** Wilcoxon rank-sum test bar plot showing significant changes in the relative abundance at the phylum level. The *p* value is shown on the right. * *p* < 0.05, ** *p* < 0.01. **e** Heatmap showing relative abundance of bacteria at the genus level. **f** Wilcoxon rank-sum test bar plot showing changes of bacteria at the genus level. The p value is shown on the right. * *p* < 0.05, ** *p* < 0.01. **g** Spearman correlation heatmap indicating the correlation of bacteria at the genus level with fasting blood glucose levels and body weight. Correlation coefficient is indicated

As the gut microbiota has been proposed to underlie the beneficial effects of fasting [13], we investigated the gut microbiota in these mice. We performed 16S rRNA gene sequencing on the collected feces. The gut microbiota of the FMD mice featured a higher Simpson index and a significant difference in α diversity when compared with the control mice (Fig. 5a), indicating an increase in the richness of the microbiome. Respective rarefaction curves became flatter to the right (Additional file 1: Figure S1), indicating that the sample community was sufficiently extrapolated and that the community of gut microbiota was fully represented.

A genus-level hierarchical clustering tree illustrated structural rearrangement of the gut microbiota after 2 months of intermittent administration of the FMD (Fig. 5b). Principal coordinate analysis (PCoA) of the relative abundance of bacteria at the genus level demonstrated a distinct compositional shift of the gut microbiome after FMD application (Fig. 5c). Wilcoxon rank-sum test revealed that at the level of the phyla, the FMD increased *Bacteroidetes* and reduced *Firmicutes* and *Saccharbacteria* (Fig. 5d). Community heatmap analysis at the genus level showed alterations in numerous gut bacteria after the FMD (Fig. 5e). Further analysis using Wilcoxon rank-sum tests demonstrated a significant increase in the genera of *Parabacteroides* and *Blautia* after intermittent application of the FMD (Fig. 5f). In contrast, the FMD significantly reduced *Prevotellaceae*, *Alistipes* and *Ruminococcaceae* (Fig. 5f). At the species level, the FMD significantly increased *Bacteroidales S24–7* and *Parabacteroides distasonis* (Additional file 1: Figure S2). In addition, a few species, including *Prevotellaceae UCG-001*, *Alistipes*, *Lachnospiraceae NK4A136*, and *Ruminococcaceae UCG-014*, were significantly reduced by the FMD (Additional file 1: Figure S2A).

To further investigate whether these changes in the bacteria were correlated with the blood glucose levels and body weight, we established a Spearman correlation heatmap at genus level (Fig. 5g). *Alistipes*, *Lachnospiraceae NK4A136, Prevotellaceae UCG-001, Ruminococcaceae UCG-014*, and a few other genera were positively correlated with the fasting blood glucose levels (Fig. 5g). In contrast, *Blautia*, *Coprococcus* and *Parabacteroides* were negatively correlated with the fasting blood glucose levels (Fig. 5g). Additionally, changes in many of these bacteria were correlated with the body weight of the mice (Fig. 5g). Analysis at species level also showed that *Parabacteroides distasonis* was negatively correlated with fasting blood glucose levels (Additional file 1: Figure S2B). On the other hand, many bacteria, including *Ruminococcaceae UCG-015, Prevotellaceae UCG-001, Alistipes,* and *Lachnospiraceae NK4A136*, were positively correlated with fasting blood glucose levels (Additional file 1: Figure S2B). Collectively, these data indicated that the intermittent application of the FMD causes alterations in the gut microbiota and that some of these changes are correlated with the blood glucose levels in mice.

### Intermittent administration of FMD improves the progression of type 1 diabetes in mice

**Fig. 6:**
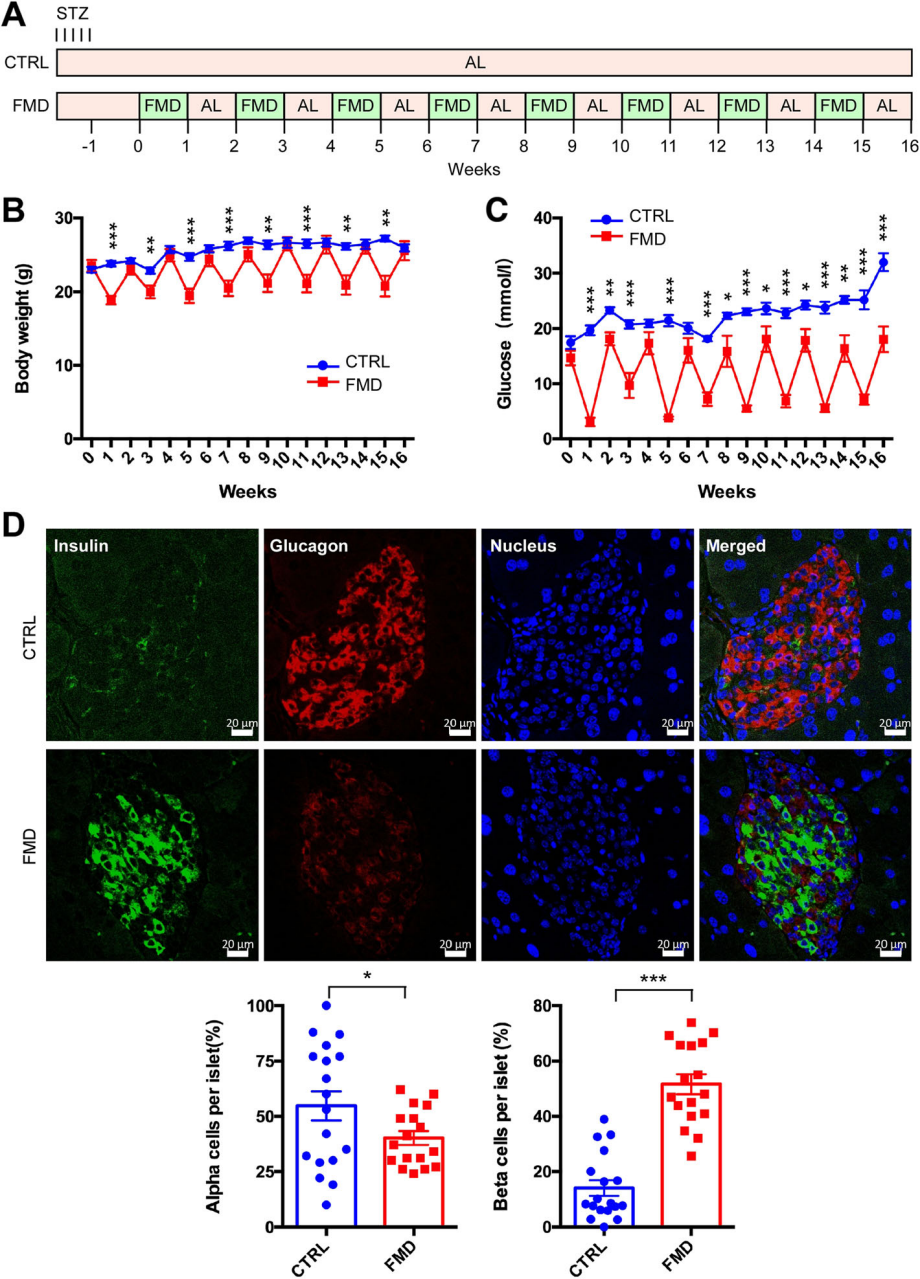
Intermittent FMD improves hyperglycemia in type 1 diabetic mouse model. **a** Experimental scheme to determine effects of the FMD in T1D in STZ-treated mice. Six-week-old male C57BL/6 mice were assigned to two groups: control and FMD (*n* = 6 for control and *n* = 7 for FMD). STZ (40 mg/kg) was administered intraperitoneally for five consecutive days. An equal volume of citrate buffer was injected into control mice. Each FMD cycle entails 7 days of FMD and 7 days of ad libitum feeding (AL). During AL period, the mice had free access to normal chow. **b** Body weight. **c** Blood glucose levels. Blood samples were collected on the last day of each cycle. Mice were fasted for 6 h (morning fasting) before glucose measurements. **d** Immunostaining of pancreatic sections to detect expression of insulin and glucagon. Scale bar: 20 μm. The percentage of β cells and α cells per islet is shown in the lower panel. Data are expressed as the mean ± SEM, * *p* < 0.05, ** *p* < 0.01, *** *p* < 0.001

To further explore the interventional effect of intermittent FMD on diabetes, we analyzed a type 1 diabetes (T1D) mouse model that was generated by STZ injection in C57BL/6 mice [21]. The mice were divided into two groups: the control group with free access to normal chow and the FMD group that took FMD (~ 1/3 cal of the control) every other week (Fig. 6a). As expected, the body weight fluctuated along with the administration of FMD (Fig. 6b). The fasting blood glucose level was significantly reduced at the end of each FMD week (Fig. 6c). Interestingly, the fasting blood glucose level at the end of AL week in the FMD group continued to be significantly lower than the control group since the 8th week (Fig. 8c), indicating that the hyperglycemia of the T1D mice was improved by intermittent FMD in later stage. Consistently, glucose tolerance as measured by GTT was significantly improved by FMD (Additional file 1: Figure S3A), while ITT was slightly improved by FMD (Additional file 1: Figure S3B). The area of the pancreatic islet was elevated by FMD as analyzed by H&E staining (Additional file 1: Figure S3C). In addition, fluorescence staining revealed that the number of islet β cells was significantly increased by FMD, together with a reduction in the number of α cells (Fig. 6d). Collectively, these data indicate that intermittent administration of FMD is able to intervene in the progression of T1D in the mice.

## Discussion

Our study is in agreement with numerous previous studies demonstrating that IF is a beneficial intervention in diabetes. IF has been found to improve glucose tolerance and insulin resistance in heterozygous BDNF knockout mice and was associated with reduction of obesity and an increase in locomotor activity [22]. IF was able to significantly improve glucose homeostasis in sand rats [23] and streptozotocin-induced diabetic rats [24]. High fat diet (HFD)-fed mice experiencing TRF consumed the same number of calories as the control groups, but TRF provided protection against obesity, hyperinsulinemia, hepatic steatosis, and inflammation together with improved motor coordination [25].

The beneficial effects of IF on health was originally proposed to be achieved by two mechanisms: reduction in oxidative damage and increase in cellular stress resistance [26]. IF was able to reduce mitochondrial generation of reactive oxygen species (ROS), indicating a beneficial antioxidant effect that could reduce oxidative stress associated with aging [27]. In addition, TRF in mice is associated with improvements in the function of CREB, mTOR and AMPK pathways, which are closely linked to metabolic homeostasis of the body [28]. IF is also associated with a reduction in inflammation, as it has been found that reduction in dietary energy intake differentially modulates neurotrophic and inflammatory pathways to protect neurons against ischemic injury [29]. Lately, autophagy has been shown to be crucial in mediating the beneficial effects on IF on diabetes. Under the condition of HFD, IF restores autophagic flux in islets and improves glucose tolerance via increasing glucose-stimulated insulin secretion, β cell survival, and nuclear expression of pancreatic regeneration marker Ngn3 [30].

Many recent studies have indicated that gut microbiota are involved in the development of type 2 diabetes [31–33]. We observed that the composition of gut microbiota was markedly altered by the FMD. Intermittent application of the FMD significantly increased the bacteria *Parabacteroides distasonis* and *Blautia*, and these bacteria were associated with lower blood glucose levels. On the other hand, the FMD decreased the levels of *Prevotellaceae UCG-001, Alistipes* and *Ruminococcaceae UCG-01,* and these bacteria were associated with higher blood glucose levels. The changes in the bacteria found in our study are consistent with the observations reported by other groups. In particular, the gut bacteria *Parabacteroides distasonis* was found to mediate the beneficial effects of the Mediterranean diet in improving insulin sensitivity in obese human subjects [34]. *Prevotellaceae* was increased in the gut microbiota of T2D patients [35]. In *ob/ob* mice, *Prevotellaceae* was associated with impaired glucose tolerance, while *Lachnospiraceae* was correlated with improvements in glucose tolerance [36]. In addition, *Blautia*, which was increased by the FMD, was found to be elevated after treatment with berberine and metformin in diabetic rats [37]. Considering the contribution of gut microbiota to the development of diabetes, it is important to investigate how alterations in the gut microbiome mediate the interventional effect of the FMD on type 2 diabetes in the future.

Our study has provided further evidence indicating that IF is able to preserve or restore β cell function, as suggested by a recent study from Dr. Longo’s group [19], in which it was found that Ngn3-driven β cell regeneration occurred after FMD application in diabetic mice. As continuous fasting has not been reported to aid in the regeneration of β cells in *db/db* mice, we propose that the pattern of intermittent fasting is key to restoring β cell function. One of the unanswered question concerns identification of the best regime of intermittent fasting. Cheng’s study used a 4-day FMD, alternating with 7 days of free feeding in *db/db* mice [19]. Our study used a 7-day FMD alternating with 7 days of free eating. Another difference between our FMD administration and that in Cheng’s study is the energy intake. In Cheng’s study, the mice took the FMD for 4 days followed by al libitum standard chow for up to 10 days [19]. When using the FMD in their study, the mice were fed 50% of the normal caloric intake on day 1 and 10% caloric intake on days 2–4 [19]. In our study, the mice were given ~ 1/3 of the caloric intake each day for 7 days, followed by al libitum standard chow for 7 days. It is important to find the optimal fasting period interval as well as the optimal caloric restriction in the future. Another unanswered question concerns the degree to which the protective effect of an intermittent FMD is due to the reduction in obesity of the mice, as numerous previous studies in both animals and humans have indicated that the reduction in obesity is closely associated with the amelioration of diabetes. Interestingly, in our study, body weight fluctuated with the FMD regime (Fig. 1c). Even at the points in which the FMD mice regained body weight at a level similar to that of the control mice, blood glucose levels were still lower in the FMD group (Fig. 1c). Such an observation favors the idea that the reduction in obesity cannot completely explain the protective effect of intermittent fasting on diabetes. We therefore propose that the improvement of β cell function in diabetic mice is mainly due to intermittent FMD rather than body weight loss.

It is worth noting that the FMD used in this study is mainly plant-based and contains hundreds of ingredients and many of them have bioactivities. At present we cannot rule out the possibility that some of the observed beneficial effects of FMD administration are caused by the bioactive molecules of the FMD in addition to intermittent fasting. Nevertheless, our study provides strong evidence to support that the intermittent administration of an FMD is an effective interventional strategy in diabetic mice, paving the way for future studies in humans.

## Additional file

Additional file 1: **Figure S1.** Rarefactions curve of gut microbiota in the two groups of mice. **Figure S2.** Intermittent FMD alters the composition of gut microbiota at species level. **Figure S3.** Improvement of glucose tolerance and islet area by FMD in type 1 diabetic mice. **Table S1.** Ingredients of FMD. **Table S2.** Nutritional information of FMD. **Table S3.** Nutritional information of normal chow. **Table S4.** Primer sequences used in RT-PCR with liver tissues. (PDF 695 kb)

## Abbreviations

Acaa1: acetyl-coenzyme A acyltransferase 1
Acox1: acyl-coenzyme A oxidase 1
*db/db* mice: C57BL/ksJ-db mice
Ehhadh: enoyl-CoA hydratase and 3-hydroxyacyl CoA dehydrogenase
FMD: Fasting-mimicking diet
HOMA: Homeostatic model assessment
IF: Intermittent fasting
PCoA: Principal coordinate analysis
PF: Periodic fasting
PPARα: Peroxisome proliferator-activated receptor α
ROS: Reactive oxygen species
STZ: Streptozotocin
TG: Total glyceride
TRF: Time-restricted feeding

## References

[1] Longo VD, Mattson MP (2014). Fasting: molecular mechanisms and clinical applications. Cell Metab.

[2] Mattson MP, Longo VD, Harvie M (2017). Impact of intermittent fasting on health and disease processes. Ageing Res Rev.

[3] Horne BD, Muhlestein JB, Anderson JL (2015). Health effects of intermittent fasting: hormesis or harm? A systematic review. Am J Clin Nutr.

[4] Buono R, Longo VD (2018). Starvation, Stress Resistance, and Cancer. Trends Endocrinol Metab.

[5] Carlson AJ, Hoelzel F (1946). Apparent prolongation of the life span of rats by intermittent fasting. J Nutr.

[6] Duncan GG, Jenson WK, Fraser RI, Cristofori FC (1962). Correction and control of intractable obesity. Practicable application of intermittent periods of total fasting. Jama.

[7] Mattson MP (2005). Energy intake, meal frequency, and health: a neurobiological perspective. Annu Rev Nutr.

[8] Bruce-Keller AJ, Umberger G, McFall R, Mattson MP (1999). Food restriction reduces brain damage and improves behavioral outcome following excitotoxic and metabolic insults. Ann Neurol.

[9] Anson RM, Guo Z, de Cabo R, Iyun T, Rios M, Hagepanos A, Ingram DK, Lane MA, Mattson MP (2003). Intermittent fasting dissociates beneficial effects of dietary restriction on glucose metabolism and neuronal resistance to injury from calorie intake. Proc Natl Acad Sci U S A.

[10] Varady KA, Dam VT, Klempel MC, Horne M, Cruz R, Kroeger CM, Santosa S (2015). Effects of weight loss via high fat vs. low fat alternate day fasting diets on free fatty acid profiles. Sci Rep.

[11] Chung H, Chou W, Sears DD, Patterson RE, Webster NJ, Ellies LG (2016). Time-restricted feeding improves insulin resistance and hepatic steatosis in a mouse model of postmenopausal obesity. Metab Clin Exp.

[12] Chaix A, Zarrinpar A, Miu P, Panda S (2014). Time-restricted feeding is a preventative and therapeutic intervention against diverse nutritional challenges. Cell Metab.

[13] Li G, Xie C, Lu S, Nichols RG, Tian Y, Li L, Patel D, Ma Y, Brocker CN, Yan T (2017). Intermittent fasting promotes white adipose Browning and Decreases obesity by shaping the gut microbiota. Cell Metab.

[14] Trepanowski JF, Kroeger CM, Barnosky A, Klempel MC, Bhutani S, Hoddy KK, Gabel K, Freels S, Rigdon J, Rood J (2017). Effect of alternate-day fasting on weight loss, weight maintenance, and Cardioprotection among metabolically healthy obese adults: a randomized clinical trial. JAMA Intern Med.

[15] Brandhorst S, Choi IY, Wei M, Cheng CW, Sedrakyan S, Navarrete G, Dubeau L, Yap LP, Park R, Vinciguerra M (2015). A periodic diet that mimics fasting promotes multi-system regeneration, enhanced cognitive performance, and Healthspan. Cell Metab.

[16] Choi IY, Piccio L, Childress P, Bollman B, Ghosh A, Brandhorst S, Suarez J, Michalsen A, Cross AH, Morgan TE (2016). A diet mimicking fasting promotes regeneration and reduces autoimmunity and multiple sclerosis symptoms. Cell Rep.

[17] Di Biase S, Lee C, Brandhorst S, Manes B, Buono R, Cheng CW, Cacciottolo M, Martin-Montalvo A, de Cabo R, Wei M (2016). Fasting-mimicking diet reduces HO-1 to promote T cell-mediated tumor cytotoxicity. Cancer Cell.

[18] Wei Min, Brandhorst Sebastian, Shelehchi Mahshid, Mirzaei Hamed, Cheng Chia Wei, Budniak Julia, Groshen Susan, Mack Wendy J., Guen Esra, Di Biase Stefano, Cohen Pinchas, Morgan Todd E., Dorff Tanya, Hong Kurt, Michalsen Andreas, Laviano Alessandro, Longo Valter D. (2017). Fasting-mimicking diet and markers/risk factors for aging, diabetes, cancer, and cardiovascular disease. Science Translational Medicine.

[19] Cheng CW, Villani V, Buono R, Wei M, Kumar S, Yilmaz OH, Cohen P, Sneddon JB, Perin L, Longo VD (2017). Fasting-mimicking diet promotes Ngn3-driven beta-cell regeneration to reverse diabetes. Cell.

[20] Tang R, Wei Y, Li Y, Chen W, Chen H, Wang Q, Yang F, Miao Q, Xiao X, Zhang H (2018). Gut microbial profile is altered in primary biliary cholangitis and partially restored after UDCA therapy. Gut.

[21] Wu KK, Huan Y. SStreptozotocin-induced diabetic models in mice and rats. Current Prot Pharmacol. 2008:47, Chapter 5, Unit 5. 10.1002/0471141755.ph0547s40. 10.1002/0471141755.ph0547s40 22294227

[22] Duan W, Guo Z, Jiang H, Ware M, Mattson MP (2003). Reversal of behavioral and metabolic abnormalities, and insulin resistance syndrome, by dietary restriction in mice deficient in brain-derived neurotrophic factor. Endocrinology.

[23] Belkacemi L, Selselet-Attou G, Louchami K, Sener A, Malaisse WJ (2010). Intermittent fasting modulation of the diabetic syndrome in sand rats. II In vivo investigations. Int J Mol Med.

[24] Belkacemi L, Selselet-Attou G, Hupkens E, Nguidjoe E, Louchami K, Sener A, Malaisse WJ (2012). Intermittent fasting modulation of the diabetic syndrome in streptozotocin-injected rats. Int J Endocrinol.

[25] Harvie MN, Pegington M, Mattson MP, Frystyk J, Dillon B, Evans G, Cuzick J, Jebb SA, Martin B, Cutler RG (2011). The effects of intermittent or continuous energy restriction on weight loss and metabolic disease risk markers: a randomized trial in young overweight women. Int J Obes.

[26] Mattson MP, Wan R (2005). Beneficial effects of intermittent fasting and caloric restriction on the cardiovascular and cerebrovascular systems. J Nutr Biochem.

[27] Descamps O, Riondel J, Ducros V, Roussel AM (2005). Mitochondrial production of reactive oxygen species and incidence of age-associated lymphoma in OF1 mice: effect OF alternate-day fasting. Mech Ageing Dev.

[28] Hatori M, Vollmers C, Zarrinpar A, DiTacchio L, Bushong EA, Gill S, Leblanc M, Chaix A, Joens M, Fitzpatrick JA (2012). Time-restricted feeding without reducing caloric intake prevents metabolic diseases in mice fed a high-fat diet. Cell Metab.

[29] Arumugam TV, Phillips TM, Cheng A, Morrell CH, Mattson MP, Wan R (2010). Age and energy intake interact to modify cell stress pathways and stroke outcome. Ann Neurol.

[30] Liu H, Javaheri A, Godar RJ, Murphy J, Ma X, Rohatgi N, Mahadevan J, Hyrc K, Saftig P, Marshall C (2017). Intermittent fasting preserves beta-cell mass in obesity-induced diabetes via the autophagy-lysosome pathway. Autophagy.

[31] Qin J, Li Y, Cai Z, Li S, Zhu J, Zhang F, Liang S, Zhang W, Guan Y, Shen D (2012). A metagenome-wide association study of gut microbiota in type 2 diabetes. Nature.

[32] Karlsson FH, Tremaroli V, Nookaew I, Bergstrom G, Behre CJ, Fagerberg B, Nielsen J, Backhed F (2013). Gut metagenome in European women with normal, impaired and diabetic glucose control. Nature.

[33] Arora T, Backhed F (2016). The gut microbiota and metabolic disease: current understanding and future perspectives. J Intern Med.

[34] Haro C, Montes-Borrego M, Rangel-Zuniga OA, Alcala-Diaz JF, Gomez-Delgado F, Perez-Martinez P, Delgado-Lista J, Quintana-Navarro GM, Tinahones FJ, Landa BB (2016). Two healthy diets modulate gut microbial community improving insulin sensitivity in a human obese population. J Clin Endocrinol Metab.

[35] Fugmann M, Breier M, Rottenkolber M, Banning F, Ferrari U, Sacco V, Grallert H, Parhofer KG, Seissler J, Clavel T (2015). The stool microbiota of insulin resistant women with recent gestational diabetes, a high risk group for type 2 diabetes. Sci Rep.

[36] Ellekilde M, Krych L, Hansen CH, Hufeldt MR, Dahl K, Hansen LH, Sorensen SJ, Vogensen FK, Nielsen DS, Hansen AK (2014). Characterization of the gut microbiota in leptin deficient obese mice - correlation to inflammatory and diabetic parameters. Res Vet Sci.

[37] Zhang X, Zhao Y, Xu J, Xue Z, Zhang M, Pang X, Zhang X, Zhao L (2015). Modulation of gut microbiota by berberine and metformin during the treatment of high-fat diet-induced obesity in rats. Sci Rep.

