# Supplementary material for: Intermittent administration of a fasting-mimicking diet intervenes in diabetes progression, restores β cells and reconstructs gut microbiota in mice
Source: Nutr Metab (Lond). 2018 Nov 20;15:80. doi: 10.1186/s12986-018-0318-3 (PMC6245873; doi:10.1186/s12986-018-0318-3)
Supplement: Supplementary file 1 — Figure S1. Rarefactions curve of gut microbiota in the two groups of mice. Figure S2. Intermittent FMD alters the composition of gut microbiota at species level. Figure S3. Improvement of glucose tolerance and islet area by FMD in type 1 diabetic mice. Table S1. Ingredients of FMD. Table S2. Nutritional information of FMD. Table S3. Nutritional information of normal chow. Table S4. Primer sequences used in RT-PCR with liver tissues. (PDF 695 kb) [file 12986_2018_318_MOESM1_ESM.pdf]

Supplemental Materials

Intermittent administration of a fasting-mimicking diet intervenes in diabetes progression, restores β cells and reconstructs gut microbiota in mice

Siying Wei, Ruomei Han, Jingyu Zhao, Shuo Wang, Meiqin Huang, Yining Wang, and Yan Chen

There are a total of 3 Supplemental Figures and 4 Supplemental Tables.

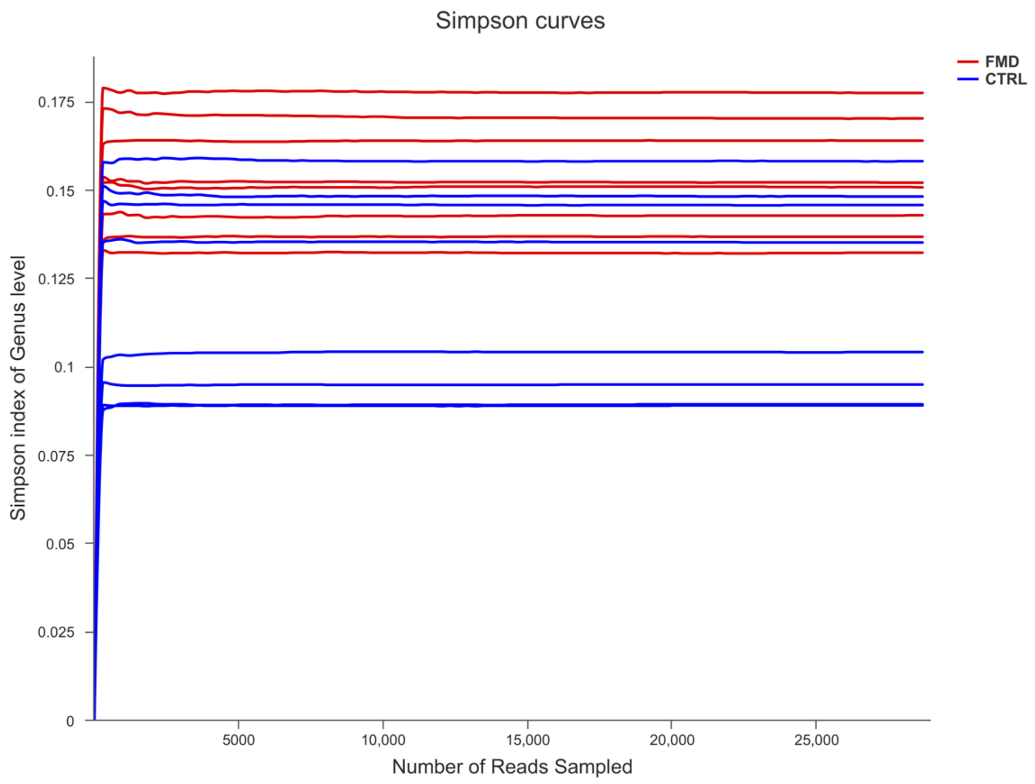

Supplemental Figure 1. Rarefactions curve of gut microbiota in the two groups of mice

Rarefactions curve Simpson index at genus level (n = 7 mice/group).

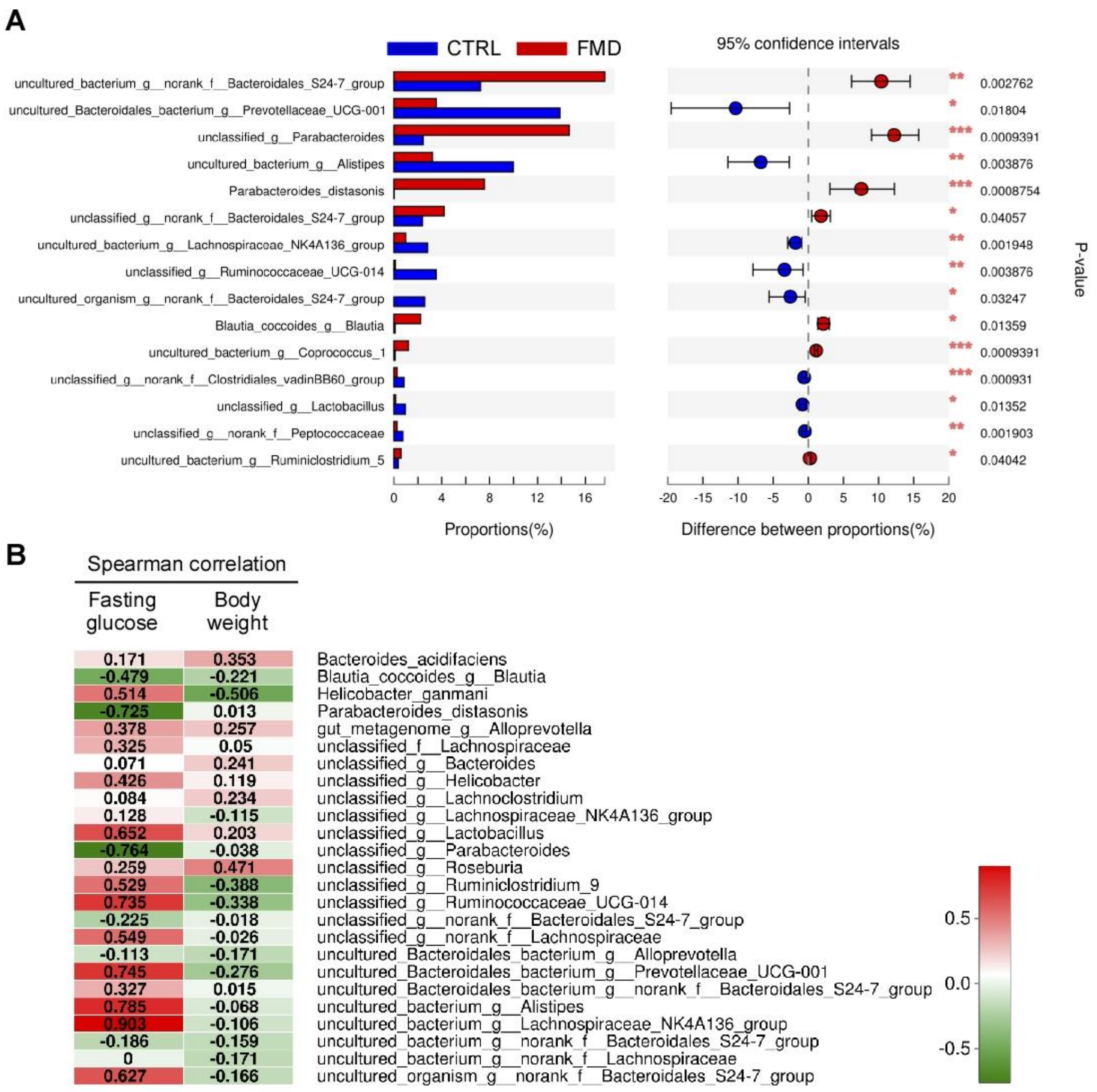

Supplemental Figure 2. Intermittent FMD alters the composition of gut microbiota at species level

(A) Wilcoxon rank-sum test bar plot showing changes of bacteria at species level. The p value is shown in the right. * $p < 0.05$, ** $p < 0.01$.

(B) Spearman correlation heatmap indicating the correlation of bacteria at species level with fasting blood glucose and body weight. Correlation coefficient is shown inside.

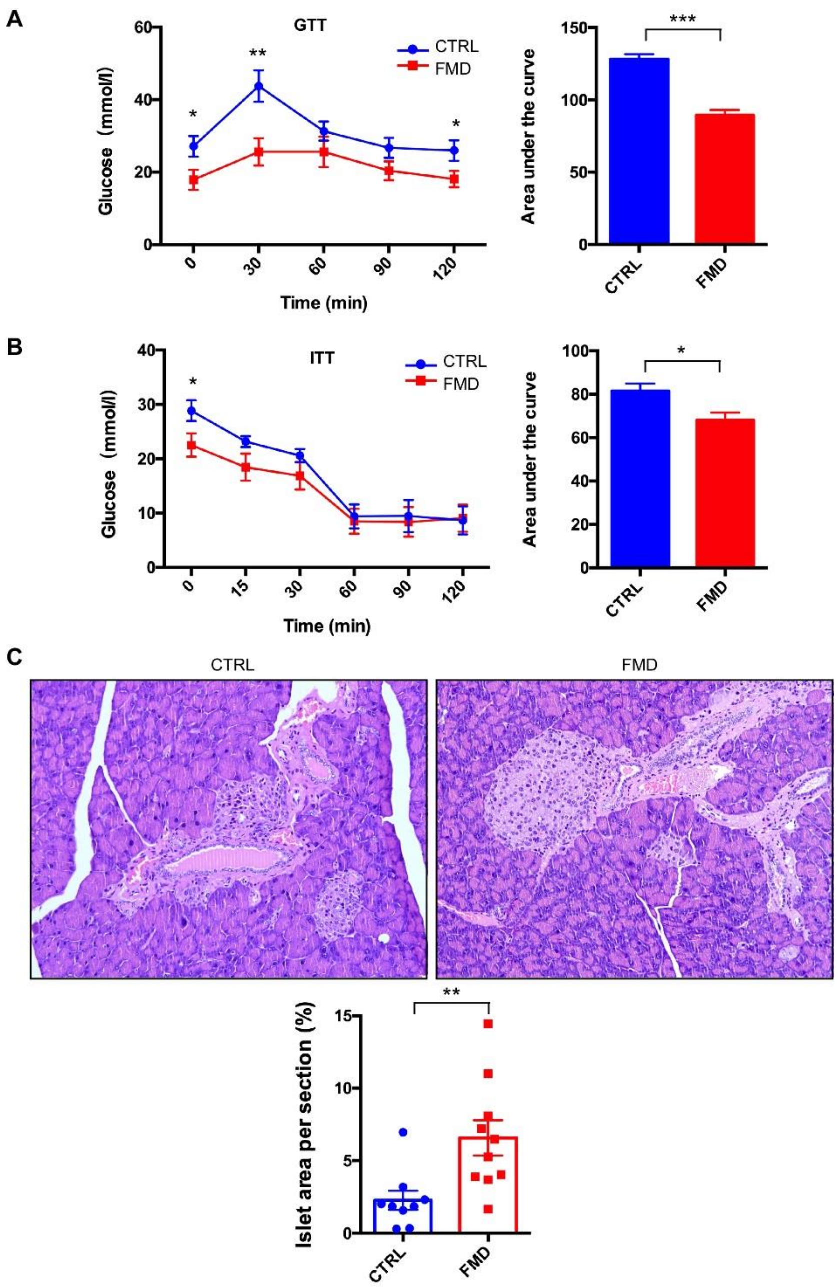

Supplemental Figure 3. Improvement of glucose tolerance and islet area by FMD in type 1 diabetic mice

(A, B) Glucose tolerance test and insulin tolerance test at the end of the expeiment. The area under the curve is shown on the right.

(C) Representative H&E staining of islets in pancreatic sections (X20). The relative value of islet area in the pancreas sections is shown in the lower panel.

Data are expressed as the mean ± SEM, * $p < 0.05$, ** $p < 0.01$, *** $p < 0.001$.

Supplemental Table 1. Ingredients of FMD

High protein high fiber crispy grain (isolated soybean protein, rice, polyglucan, calcium carbonate), Isomaltose Hypgather syrup, Chia seed, Fruit and vegetable powder (Coconut milk, Avocado, Passion fruit, Crab apple, Fig, Mango, Apple, Guava, Blueberry, Blackberry, Strawberry, Raspberry, Waxberry, Green plum, Sea buckthorn, Coconut, Wax apple, Loquat, Mulberry, Lychee, Pawpaw, Peach, Kiwi, Carambola, Cherry, Orange, Grapefruit, Citrus, Grape, Apricot, Kumquat, Plum, Persimmon, Banana, Pineapple, Lemon, Hamimelon, Red sugarcane, Pear, Melon, Watermelon, Fermented pumpkin, Fermented carrot, Spinach, Asparagus, Red tomato, Cherry tomato, Eggplant, White gourd, Chervil, Garland chrysanthemum, Broccoli, Leaf mustard, Cabbage, Lettuce, Sweet pepper, Mustard, Pumpkin, Cucumber, White radish, Bitter gourd, Black potato, Lotus root, Alfalfa, Lettuce, Chinese cabbage, Kale, Turnip cabbage, Purple kale, Green kale, Okra, Black tomato, Red pepper, Purple onion, Rape, Coriander, Taro, Sweet potato, Wheat seedlings, Barley seedlings, Brown rice, Glutinous rice, Wheat, Barley, Oats, Rye, Black rice, Black corn, Sorghum, Highland barley, Millet, Buckwheat, Pearl barley, Quinoa, Red beans, Mung beans, Green beans, Pinto beans, Garden peas, Purple lentils, Cowpea, Chickpea, Green soy bean, Black lentils, White lentils, Dutch beans, Green beans), Glycerin, White chocolate, High oleic acid sunflower seed oil, Resistance dextrin (Water-soluble dietary fiber), Salted vegetable grain, Cranberry grain, Nutrition powder (Flaxseed, Bitter buckwheat, Oat fiber, Psylliumseedhusk, Conjugated linoleic acid glyceride, Medium chain glycerin triglyceride, Perilla oil, Yeast extract, Raw coffee, Potato extract, Chitosan oligosaccharide, Ginger (black), L-Arabic sugar, Seaweed, Cactus, Nutritional yeast, Yeast-beta-glucan, Beta-carotene, Lutein ester, Lycopene, Fish oil extract, Milk mineral salt, Tianshan lake salt (cooking salt), Walnut peptide, Bitter gourd peptide, Gelatin peptide, Albumin peptide, Collagen peptide, Pea peptide), Grain oats, Red bean and pearl barley

grain, High fiber brittle grain, Oligofructose liquid, Calcium caseinate, Milk protein powder, Concentrate whey protein powder, Separate whey protein powder, Hydrolyze whey protein powder, Frozen dried strawberry granule, Frozen Dried purple sweet potatoes, Frozen Dried mango, Whole milk powder, Flavor yoghourt powder, Almond grain, White sesame seed, Hazelnut, Konjaku flour, Kiwi powder, Phospholipid, Polydextrose, Nut powder (Peanut, Sunflower kernels, Pumpkin seed kernels, Walnut meat, Pistachio nuts, Chestnut, Pine nuts, Macadamia nuts and Almonds), Mushroom powder (Shiitake, Golden mushroom, Oyster mushroom, King oyster mushroom, Monkey mushroom, Tea tree mushroom, Flower mushroom, Straw mushroom, Coprinus comatus, Tremella, Snake butter, Matsutake, Truffle, bisporus, black fungus, Agaricus bisporus), Algae products (Laver, Kelp, Undaria, Spiral seaweed), Herb powder (Amaranth rapeseed, Coriander seeds, Celery seeds, Rapeseed, Cucumber seeds, Grape seeds, Watermelon seeds, Lettuce seeds, Potherb mustard, Flatmelon seeds, Bitter gourd seeds, Cucurbita seeds, Radish seeds, Cauliflower seeds, Endive seeds, Pomegranate seeds, Chinese cabbage seeds, Spinach seeds, Carrot seeds, Garland chrysanthemum seeds, Perilla seeds, Double red rose, Honeysuckle, Osmanthus flower, Seville orange flower, White lentil flower, Sophora flower, Lilac, Lily, Ginkgo, Jack bean, Hawthorn, Semen cassia seed, Gordon fruit, Poria, Chinese yam, Pueraria lobata, Platycodon, Lotus leaf, Polygonatum, Lotus seed, Dark plum, Coix seed, Raspberry, Thistle, Purslane, Polygonatum odoratum, Muscade, Emblic leaf flower fruit, Fingered citron, Chinese gooseberry, Chinese olive, Cordate houttuynia, Licorice, Gardenia, Boat-fruited scaphium seed, Citron, Chinese mosla herb, Folium mori, Vermilion, Lophatherum gracile, Fermented soybean, Witloof, Sophora flower bud, Dandelion, Couch grass root, Reed rhizome, Orange peel, Peppermint, Patchouli), Probiotics (Lactobacillus Bifidobacterium, Plant Lactobacillus, Lactobacillus acidophilus), Vitamin A, Vitamin D, Vitamin E, Vitamin B_1, Vitamin B_2, Vitamin B_6, Vitamin B_{12}, Vitamin C, Niacin, Folic acid, Pantothenic acid, Calcium carbonate, Pyrophosphate, Zinc gluconate.

Supplemental Table 2. Nutritional information of FMD

Item	Per 100g
Energy	1681KJ
Protein	17.8g
Fat	14.2g
Carbohydrate	44.3g
Dietary fiber	12.5g
Sodium	260mg
Vitamin A	301ug
Vitamin D	2.2ug
Vitamin E	8.05mg
Vitamin B1	1.07mg
Vitamin B2	1.05mg
Vitamin B6	1.02mg
Vitamin B12	0.66ug
Vitamin C	45.9mg
Niacin acid	8.10mg
Folic acid	171ug
Pantothenic acid	3.00mg
Phosphorus	325mg
Potassium	268mg
Magnesium	85mg
Calcium	380mg
Iron	6.6mg
Zinc	5.57mg
Selenium	10.0ug

Copper	0.50mg
Fluorine	0.10mg
Manganese	1.00mg

Supplemental Table 3. Nutritional information of normal chow

Item	Per 100g
Energy	1431KJ
Protein	21.59g
Fat	4.54g
Carbohydrate	51.36g
Dietary fiber	2.89g
Sodium	250mg
Vitamin A	1.6mg
Vitamin D	0.02mg
Vitamin E	29.8mg
Vitamin B1	2.9mg
Vitamin B2	3mg
Vitamin B6	3.6mg
Vitamin B12	0.0026ug
Niacin acid	18.5mg
Folic acid	1.3mg
Pantothenic acid	7.3mg
Phosphorus	910mg
Potassium	800mg
Magnesium	190mg
Calcium	1.23g
Iron	23.2mg
Zinc	6.6mg
Selenium	0.038mg
Copper	2.32mg

Manganese	10.9mg

Supplemental Table 4. Primer sequences used in RT-PCR with liver tissues

	Forward primer	Reverse primer
mus-Acox1	TAACTTCCTCACTCGAAGCCA	AGTTCCATGACCCATCTCTGTC
mus-Fgf21	CTGCTGGGGGTCTACCAAG	CTGCGCCTACCACTGTTCC
mus-Ehhadh	ATGGCTGAGTATCTGAGGCTG	GGTCCAAACTAGCTTTCTGGAG
mus-Cd36	ATGGGCTGTGATCGGAACTG	GTCTTCCCAATAAGCATGTCTCC
mus-Scd1	GCGATACACTCTGGTGCTCA	CCCAGGGAAACCAGGATATT
mus-Acaa1b	ATGCTTCCATGCTGAGATTGT	TCCATCCTTGAAGGCAGGCTT
mus-Fas	AGGTGGTGATAGCCGGTATGT	TGGGTAATCCATAGAGCCCAG
mus-Acc1	TGACAGACTGATCGCAGA-GAAAG	TGGAGAGCCCCACACACA
mus-Srebp1c	GGAGCCATGGATTGCACATT	GGCCCGGGAAGTCACTGT
mus-Chrebp	CCTCACTTCACTGTGCCTCA	ACAGGGGTTGTTGTCTCTGG
mus-Fabp	ATGAACTTCTCCGGCAAGTACC	GGTCCTCGGGCAGACCTAT
mus-ApoB	CGTGGGCTCCAGCATTCTA	TCACCAGTCATTTCTGCCTTTG
mus-ApoE	GCTGGGTGCAGACGCTTT	TGCCGTCAGTTCTTGTGTGACT
mus-Actin	GATCATTGCTCCTCCTGAGC	ACTCCTGCTTGCTGAT CCAC
mus-IL-1β	GAAGAAGAGCCCATCCTCTG	TCATCTCGGAGCCTGTAGTG
mus-IL-6	GGACCAAGACCATCCAATTC	ACCACAGTGAGGAATGTCCA
mus-TNF-α	GACAGTGACCTGGACTGTGG	TGAGACAGAGGCAACCTGAC
